# A study on *q*-analogue of generalized Motzkin sequence spaces, their matrix transformations and compact operators

**DOI:** 10.1371/journal.pone.0329210

**Published:** 2025-08-20

**Authors:** Jun-Jie Quan, Devia Narrania, Kuldip Raj, Qing-Bo Cai

**Affiliations:** 1 Department of Mathematics and Digital Sciences, Chengyi College, Jimei University, Xiamen, Fujian, China; 2 School of Mathematics, Shri Mata Vaishno Devi University, Katra, Jammu and Kashmir, India; 3 School of Mathematics and Computer Science, Quanzhou Normal University, Quanzhou, China; Sultan Qaboos University, OMAN

## Abstract

In this article, we have constructed generalized *q*-difference Motzkin sequence spaces $M_{0}^{q}(\Delta_{q}^{m})$, $M_{c}^{q}(\Delta_{q}^{m})$, $M_{p}^{q}(\Delta_{q}^{m})$ and $M_{\infty }^{q}(\Delta_{q}^{m})$ by composing *q*-Motzkin matrix with generalized *q*-difference matrix in the spaces $c_{0},c,l_{p},l_{\infty }$, respectively and explore their topological properties. We determine the bases for $M_{c}^{q}(\Delta_{q}^{m})$ and $M_{0}^{q}(\Delta_{q}^{m})$ and compute *α*-, *β*- and *γ*-duals of the newly defined spaces. Further, we characterize some class of matrix mappings from the spaces $M_{0}^{q}(\Delta_{q}^{m})$ and $M_{c}^{q}(\Delta_{q}^{m})$ to the spaces $c,c_{0},cs,cs_{0},l_{\infty },l_{1},bs$. Lastly, compact operators are characterized on the spaces $M_{0}^{q}(\Delta_{q}^{m})$ using Hausdorff measure of noncompactness.

## Introduction

Sequence spaces have played a vital role across various branches of mathematics, such as functional analysis, operator theory, and approximation theory. The importance of sequence spaces has sparked considerable interest among researchers in summability theory. They have introduced and investigated different types of sequence spaces to uncover their unique properties. For example $l_{\infty },\;l_{p},\;c,\;c_{0},$ denote the spaces of all bounded, *p*-summable, convergent and null sequences, respectively. Further, *cs*,*cs*_0_,*bs* denote the spaces of all convergent, null and bounded series, respectively. Throughout the paper, we will denote N, R and C as the set of all natural, real and complex numbers, respectively. A sequence space *X* is called an FK-space if it is a complete linear metric space with continuous coordinates $p_{n}:X\to C$ and $p_{n}(x)=x_{n}$ for all $x=(x_{k})\in X,n\in N$, and a normed FK-space is called a BK-space. For example, *c*,*c*_0_ and $l_{\infty }$ are BK spaces with the norm $\left\|x\|=\sup_{r\in N}|x_{r}\right|$. Also, *l*_*p*_ is a BK-space with the norm defined by $\|x{\|}_{l_{p}}={\left(\sum_{k=0}^{\infty }|x_{k}{|}^{p}\right)}^{\frac{1}{p}},1\leq p<\infty .$

Let ω,Ψ denote the set of all real sequences and the set of all finite sequences, respectively. An FK-space X⊃Ψ is said to have AK if every $x=(x_{k})\in X$ has a unique representation $x=\sum_{k=0}^{\infty }x_{k}e^{\left(k\right)},$ where ${\text{e}}^{\left(\text{k}\right)}$ is the sequence whose only non-zero term is 1 in the ${\text{k}}^{\text{th}}$ place for each k∈N [1]. The spaces *c*_0_ and *l*_*p*_ have AK [2].

The primary objective of classical theory revolves around the generalization of convergence concepts for both series and sequences. Its main goal is to provide a framework through which limits can be assigned to series and sequences that do not exhibit convergence. This is achieved through the use of transformations defined by infinite matrices. The preference for utilizing matrices, rather than general linear mappings, is based on the fact that a linear mapping between two sequence spaces can be represented by an infinite matrix.

Let *Z* and *W* be two sequence spaces and $P=\left(a_{nk}\right)$ be an infinite matrix of real or complex numbers *a*_*nk*_, for $n,k\in {\mathbb{N}}_{0}=N\;\cup \;\{0\}$. Then P defines a matrix mapping from *Z* to *W*, if $Px=((Px{)}_{n})\in W$, for every sequence $x=(x_{k})\in Z$, where

$$(Px{)}_{n}=\sum_{k=0}^{\infty }a_{nk}x_{k},\;\;\;\text{where}\;n\in N_{0}.$$ (1)

The set of all these matrices is represented by the notation (*Z*,*W*). A sequence (*x*_*k*_) is P−summable to *L* if the sequence Px converges to *L*. We say that P maps *Z* regularly into *W* if $\lim_{k}x_{k}=\lim Px,$ $\forall \;(x_{k})\in Z$ and we denote the space of such matrices by (*Z*,*W*)_*reg*_.

The matrix domain $X_{P}$ of matrix P in the space *X* is a sequence space which is defined by


$$X_{P}=\{x\in \omega :Px\in X\}.$$

It holds considerable importance in the development of new sequence spaces. Moreover, if P is a triangle and *X* is a BK-space then $X_{P}$ is a BK-space. Many researchers have used this idea to construct new Banach sequence spaces by applying it to special triangles. To know about these sequence spaces, one can see [3–9].

Quantum calculus, often denoted as *q*-calculus, is a crucial mathematical tool that goes beyond traditional calculus. It plays a transformative role at the intersection of mathematics and physics. For the first time, relations between these topics especially quantum calculus (*q*-calculus) and *q*-differential operators were studied by Jackson in [10]. It has a lot of applications in different mathematical areas such as: orthogonal polynomials, hyper-geometric functions, number theory, complex analysis, combinatorics, matrix summability, approximation theory, quantum physics, particle physics, the theory of relativity, etc. For instance, Demiriz and Şahin [11] and Yaying et al. [12] developed sequence spaces using *q*-Cesàro matrix. Additionally, Yaying et al. [13] studied (*p*,*q*) analogue of Euler sequence spaces. Recently, Atabey et al. [14] developed *q*-Fibonnaci sequence spaces, Ellidokuzoglu and Demiriz [15] constructed *q*-difference sequence spaces of order *m*. For more work on *q*-sequence spaces, one can see [16–20] and references therein. One can see the basic notations on *q*-calculus in [21]. For *q*>0 and any positive integer *a*, a *q*-number is defined by


$$[a]=[a{]}_{q}=\{\begin{array}{ll} \frac{1-q^{a}}{1-q}, & \text{ if }q\neq 1,\\ a, & q=1. \end{array}$$

For the integers 0≤k≤a, *q*–binomial coefficients are defined by


(ak)q=[a]q![k]q![a−k]q!,

where the *q*–factorial [*a*]_*q*_! of *a* is given by


$$[a{]}_{q}!=\{\begin{array}{ll} {}[a][a-1]...[1], & a\geq 1,\\ 1, & a=0. \end{array}$$

The *q*-difference operator $\Delta_{q}(v)$ of $v=(v_{0},v_{1},v_{2},\ldots )$ is defined by


$$\Delta_{q}(v)=(v_{0}-v_{1},q(v_{1}-v_{2}),q^{2}(v_{2}-v_{3}),q^{3}(v_{3}-v_{4}),\ldots )$$

and the generalized *q*-difference operator $\Delta_{q}^{m}$ is defined by


(Δqmv)i,j=qmj∑i=jm(−1)i−jq(i−j2)(mi−j)qvi=qmj∑i=0m(−1)iq(i2)(mi)qvi+j.

The generalized *q*-difference matrix $\Delta_{q}^{m}=(\delta_{q}^{m}{)}_{ij}$ is given by


(δqm)ij={(−1)i−jq(i−j2)(mi−j)q,0≤j≤i0,j>i

and its inverse ${\left(\Delta_{q}^{m}\right)}^{-1}=(\delta_{q}^{m}{)}_{ij}^{-1}$ is given by


(δqm)ij−1={(−1)i−jq(i−j)(i−j−m)−(i−j2)(mi−j)q=(m+j−i−1j−i)q,0≤j≤i0,j>i.

Motzkin numbers, named after Theodore Motzkin, are a remarkable sequence of integers. In mathematics, the ${\text{r}}^{\text{th}}$ Motzkin number represents the count of distinct chords that can be drawn between *r* points on a circle without intersecting. It is important to note that the chords do not necessarily have to touch all the points on the circle.

Motzkin numbers, denoted as $M_{r}(r\in {\mathbb{N}}_{0})$, find diverse applications in various mathematical fields such as geometry, combinatorics, and number theory. They possess a recursive nature and hold significant combinatorial properties, which make them valuable tools in multiple areas of mathematics, algorithmic analysis, and even practical applications like coding theory. The Motzkin numbers have proven to be a rich source of mathematical exploration and have contributed to the understanding of fundamental concepts in different disciplines. They are represented by the following sequence:


1,1,2,4,9,21,51,127,323,835,2188,5798,15511,41835,…

The Motzkin numbers satisfy the recurrence relations


$$M_{r}=M_{r-1}+\sum_{s=0}^{r-2}M_{s}M_{r-s-2}=\frac{2r+1}{r+2}M_{r-1}+\frac{3r-3}{r+2}M_{r-2}.$$

Another relation provided by the Motzkin numbers is given below:


$$M_{r+2}-M_{r+1}=\sum_{s=0}^{r}M_{s}M_{r-s},\text{ for }r\geq 0.$$

For more detail on Motzkin numbers one can refer to [22]. Erdem et al. [23] defined the Motzkin matrix $M=(m_{rs})$ as


$${𝔪}_{rs}=\left\{ \begin{array}{cc} \frac{M_{s}M_{r-s}}{M_{r+2}-M_{r+1}} & ,0\leq s\leq r\\ 0 & ,s>r, \end{array}\right.$$

for all r,s∈ℕ and its inverse ${ℳ}^{-1}=\left({𝔪}_{rs}^{-1}\right)$ is given as


$${𝔪}_{rs}^{-1}=\left\{ \begin{array}{cc} (-1{)}^{r-s}\frac{M_{s+2}-M_{s+1}}{M_{r}}P_{r-s} & ,0\leq s\leq r\\ 0 & ,s>r, \end{array}\right.$$

where *P*_0_ = 1 and


$$P_{r}=\left|\begin{array}{cccccc} M_{1} & M_{0} & 0 & 0 & \cdots & 0\\ M_{2} & M_{1} & M_{0} & 0 & \cdots & 0\\ M_{3} & M_{2} & M_{1} & M_{0} & \cdots & 0\\ \vdots & \vdots & \vdots & \ddots & \ddots & \vdots \\ M_{r} & M_{r-1} & M_{r-2} & M_{r-3} & \cdots & M_{1} \end{array}\right|,$$

for all r∈ℕ.

Motivated by the aforementioned works on *q*-calculus, the application of *q*-difference operators, and Motzkin numbers in various mathematical and scientific disciplines, in section 2, we construct generalized Motzkin sequence spaces ${ℳ}_{0}^{q}(\Delta_{q}^{m})$, ${ℳ}_{c}^{q}(\Delta_{q}^{m})$, ${ℳ}_{p}^{q}(\Delta_{q}^{m})$, and ${ℳ}_{\infty }^{q}(\Delta_{q}^{m})$ using *q*-difference operators. Section 3 explores some topological properties and establishes the Köthe duals of the spaces ${ℳ}_{0}^{q}(\Delta_{q}^{m})$, ${ℳ}_{c}^{q}(\Delta_{q}^{m})$, ${ℳ}_{p}^{q}(\Delta_{q}^{m})$, and ${ℳ}_{\infty }^{q}(\Delta_{q}^{m})$. Section 4 presents theorems and corollaries related to matrix transformations from the spaces ${ℳ}_{0}^{q}(\Delta_{q}^{m})$ and ${ℳ}_{c}^{q}(\Delta_{q}^{m})$ into the classical sequence spaces $\ell_{\infty },c,c_{0},\ell_{1},bs,cs,cs_{0}$. Section 5 investigates the compactness of certain operators defined on the space ${ℳ}_{0}^{q}(\Delta_{q}^{m})$. Finally, Section 6 summarizes the main findings of the manuscript.

## Some new sequences spaces

We proceed by introducing *q*-Motzkin matrix $M^{q}=(m_{rs}^{q})$ as follows:


$${𝔪}_{rs}^{q}=\left\{ \begin{array}{cc} q^{s}\frac{M_{s}(q)M_{r-s}(q)}{M_{r+2}(q)-M_{r+1}(q)} & ,0\leq s\leq r\\ 0 & ,s>r, \end{array}\right.$$

for all r,s∈ℕ.

Now, using the generalized *q*-difference matrix and *q*-Motzkin matrix, we define the generalized *q*-difference Motzkin sequence spaces $M_{0}^{q}(\Delta_{q}^{m})$, $M_{c}^{q}(\Delta_{q}^{m})$, $M_{p}^{q}(\Delta_{q}^{m})$ and $M_{\infty }^{q}(\Delta_{q}^{m})$ as follows:


Mcq(Δqm)={v=(vs)∈ω:limr→∞∑s=0rq(m+1)sMs(q)Mr−s(q)Mr+2(q)−Mr+1(q)∑i=0s(−1)iq(i2)(mi)qvi+s exists },

M0q(Δqm)={v=(vs)∈ω:limr→∞∑s=0rq(m+1)sMs(q)Mr−s(q)Mr+2(q)−Mr+1(q)∑i=0s(−1)iq(i2)(mi)qvi+s=0},

Mpq(Δqm)={v=(vs)∈ω:∑r=0∞|∑s=0rq(m+1)sMs(q)Mr−s(q)Mr+2(q)−Mr+1(q)∑i=0s(−1)iq(i2)(mi)qvi+s|p<∞}

and


M∞q(Δqm)={v=(vs)∈ω:supr∈N0|∑s=0rq(m+1)sMs(q)Mr−s(q)Mr+2(q)−Mr+1(q)∑i=0s(−1)iq(i2)(mi)qvi+s|<∞}.

Let *u* = (*u*_*r*_) be the $M^{q}(\Delta_{q}^{m})-$transform of a sequence $v=(v_{s})$, which is given by the expression:

ur=(Mq(Δqm)v)r=∑s=0rqsMs(q)Mr−s(q)Mr+2(q)−Mr+1(q)qms∑i=0s(−1)iq(i2)(mi)qvi+s, (2)

for all $r\in N_{0}.$ Define *P*_0_(*q*) = 1 and


$$P_{v}(q)=\left|\begin{array}{cccccc} M_{1}(q) & M_{0}(q) & 0 & 0 & \cdots & 0\\ M_{2}(q) & M_{1}(q) & M_{0}(q) & 0 & \cdots & 0\\ M_{3}(q) & M_{2}(q) & M_{1}(q) & M_{0}(q) & \cdots & 0\\ \vdots & \vdots & \vdots & \vdots & \vdots & \vdots \\ M_{r}(q) & M_{r-1}(q) & M_{r-2}(q) & M_{r-3}(q) & \cdots & M_{1}(q) \end{array}\right|,$$

for all v∈ℕ.

Then, using Eq (2), we have

vs=∑j=0s(−1)s−jMj+2(q)−Mj+1(q)Ms(q)Ps−j(q)qs(m+s−j−1s−j)quj, (3)

for each $s\in N_{0}.$ Throughout the paper, *u* and *v* are related by Eq (2), or equivalently by Eq (3).

**Theorem 2.1.**

*1.* $M_{c}^{q}(\Delta_{q}^{m})$, $M_{0}^{q}(\Delta_{q}^{m})$ *and* $M_{\infty }^{q}(\Delta_{q}^{m})$ *are BK-spaces endowed with the norm defined by* $\|v{\|}_{M_{c}^{q}(\Delta_{q}^{m})}=\|v{\|}_{M_{0}^{q}(\Delta_{q}^{m})}=\|v{\|}_{M_{\infty }^{q}(\Delta_{q}^{m})}$
  =supr∈N0|∑s=0rq(m+1)sMs(q)Mr−s(q)Mr+2(q)−Mr+1(q)∑i=0s(−1)iq(i2)(mi)qvi+s|.
*2.* $M_{p}^{q}(\Delta_{q}^{m})$ *is a BK-spaces endowed with the norm defined by*
  ‖v‖Mpq(Δqm)=(∑r=0∞|∑s=0rq(m+1)sMs(q)Mr−s(q)Mr+2(q)−Mr+1(q)∑i=0s(−1)iq(i2)(mi)qvi+s|p)1p.

*Proof:* The sequence spaces $l_{p},l_{\infty },c,c_{0}$ are *BK*–spaces with their natural norms and $M^{q}(\Delta_{q}^{m})$ is a triangle matrix. Thus, (*i*) and (*ii*) follows immediately by using Wilansky’s work [24]. □

**Theorem 2.2.** *The spaces $M_{c}^{q}(\Delta_{q}^{m})$, $M_{0}^{q}(\Delta_{q}^{m})$, $M_{p}^{q}(\Delta_{q}^{m})$ and $M_{\infty }^{q}(\Delta_{q}^{m})$ are linearly isomorphic to c,* *c*_*0*_, *l*_*p*_ *and $l_{\infty },$ respectively.*

*Proof:* We only prove this Theorem for the space $M_{0}^{q}(\Delta_{q}^{m})$ and *c*_0_. Define the mapping $S:M_{0}^{q}(\Delta_{q}^{m})\to c_{0}$ by $S(u)=M^{q}(\Delta_{q}^{m})u,$ for all $u\in M_{0}^{q}(\Delta_{q}^{m})$ is invertible which implies that *S* is a norm preserving linear bijection. Hence, $M_{0}^{q}(\Delta_{q}^{m})≅c_{0}.$ □

**Definition 2.1.** *A sequence $v=(v_{s})\in X$ is called a Schauder basis for a normed space (X,‖.‖), if for every y∈X, there is a unique sequence of scalars $\left(\alpha_{s}\right)$ such that*


$$\lim_{n\to \infty }\|y-\sum_{s=0}^{n}\alpha_{s}v_{s}\|=0.$$

Now, we construct bases for the spaces $M_{c}^{q}(\Delta_{q}^{m})$ and $M_{0}^{q}(\Delta_{q}^{m})$. We recall that the matrix domain $X_{P}$ has a basis if and only if *X* has a basis. This statement together with Theorem 2.2 gives us the following result:

**Theorem 2.3.** *Let $u_{s}=(M^{q}(\Delta_{q}^{m})v{)}_{s}$, for all $s\in N_{0}$. For every fixed $s\in N_{0},$ define the sequence $\xi^{\left(s\right)}(q)=(\xi_{r}^{\left(s\right)}(q))$ of the elements of the space $M_{0}^{q}(\Delta_{q}^{m})$ by*


ξr(s)(q)={(−1)r−sMs+2(q)−Ms+1(q)Mr(q)Pr−s(q)qr(m+r−s−1r−s)q,0≤s≤r0,s>r.

*Then*

*1.* *the set $\left\{\xi^{\left(0\right)}(q),\xi^{\left(1\right)}(q),\xi^{\left(2\right)}(q),\ldots \right\}$ forms the basis for the space $M_{0}^{q}(\Delta_{q}^{m})$ and every $v\in M_{0}^{q}(\Delta_{q}^{m})$ has a unique representation $v=\sum_{s=0}^{\infty }u_{s}\xi^{\left(s\right)}(q).$*
*2.* *the set $\left\{e,\xi^{\left(0\right)}(q),\xi^{\left(1\right)}(q),\xi^{\left(2\right)}(q),\ldots \right\}$ forms the basis for the space $M_{c}^{q}(\Delta_{q}^{m})$ and every $v\in M_{c}^{q}(\Delta_{q}^{m})$ has a unique representation of the form $v=we+\sum_{s=0}^{\infty }(u_{s}-w)\xi^{\left(s\right)}(q),$ where $w=\lim_{s\to \infty }u_{s}=\lim_{s\to \infty }(M^{q}(\Delta_{q}^{m})v{)}_{s}.$*

*Proof:* 1. Clearly, $M^{q}(\Delta_{q}^{m})(\xi_{r}^{\left(s\right)}(q))=(e_{s})\in c_{0},$ where (*e*_*s*_) is the sequence with 1 in the ${\text{s}}^{\text{th}}$ place and zeros elsewhere for each s∈N. Now for $v\in M_{0}^{q}(\Delta_{q}^{m})$ and n∈N, we define

$$v^{\left(n\right)}=\sum_{s=0}^{n}u_{s}\xi^{\left(s\right)}(q).$$ (4)

By applying $M^{q}(\Delta_{q}^{m})$ to Eq (4), we have


$$M^{q}(\Delta_{q}^{m})v_{r}^{\left(n\right)}=\sum_{s=0}^{n}u_{s}(M^{q}(\Delta_{q}^{m})\xi_{r}^{\left(s\right)}(q))=\sum_{s=0}^{n}u_{s}e_{s}.$$

Also,


$$M^{q}(\Delta_{q}^{m})(v_{r}-v_{r}^{\left(n\right)}{)}_{s}=\left\{ \begin{array}{cc} (M^{q}(\Delta_{q}^{m})v_{r}{)}_{s} & ,s>n\\ 0 & ,0\leq s\leq n. \end{array}\right.$$

Let ϵ>0 be arbitrary. We choose $n_{0}\in N$ such that


$$|(M^{q}(\Delta_{q}^{m})v_{r}{)}_{s}|<\frac{\epsilon }{2},\;\forall \;s\geq n_{0}.$$

Then, we have


$$\left\|v-v^{\left(n\right)}{\|}_{M_{0}^{q}(\Delta_{q}^{m})}=\underset{s\geq n}{\sup}\|(M^{q}(\Delta_{q}^{m})v_{r}{)}_{s}\right\|$$

$$\leq \underset{s\geq n_{0}}{\sup}\|(M^{q}(\Delta_{q}^{m})v_{r}{)}_{s}\|$$

$$<\frac{\epsilon }{2}<\epsilon .$$

This implies $v=\sum_{s=0}^{\infty }u_{s}\xi^{\left(s\right)}(q).$

To show the uniqueness of this representation, let us assume that there exists $v=\sum_{s=0}^{\infty }w_{s}\xi^{\left(s\right)}(q).$ By the continuity of *S* transformation defined in the proof of the Theorem 2.2, we get


$$(M^{q}(\Delta_{q}^{m})v_{r}{)}_{s}=\sum_{s=0}^{\infty }w_{s}(M^{q}(\Delta_{q}^{m})\xi_{r}^{\left(s\right)}(q){)}_{s}$$

$$=\sum_{s=0}^{\infty }w_{s}(e_{s}{)}_{s},$$

which is a contradiction with the assumption that $u_{s}=(M^{q}(\Delta_{q}^{m})v_{r}{)}_{s}$ for each s∈N. Hence, the representation $v=\sum_{s=0}^{\infty }u_{s}\xi^{\left(s\right)}(q)$ is unique.

2. In a similar manner as in (i), one can easily prove (ii). □

**Theorem 2.4.** $l_{\infty }\subset M_{\infty }^{q}(\Delta_{q}^{m})$.

*Proof:* Let $v\in l_{\infty }$. Then


‖v‖M∞q(Δqm)=supr∈N0|∑s=0rq(m+1)sMs(q)Mr−s(q)Mr+2(q)−Mr+1(q)∑i=0s(−1)iq(i2)(mi)qvi+s|

≤‖v‖∞supr∈N0|∑s=0rq(m+1)sMs(q)Mr−s(q)Mr+2(q)−Mr+1(q)∑i=0s(−1)iq(i2)(mi)q|

$$=\|v{\|}_{\infty }<\infty .$$

Thus, $v\in M_{\infty }^{q}(\Delta_{q}^{m}).$ □

## *α*-, *β*- and *γ*-duals

In this section, we compute *α*-, *β*- and *γ*-duals of the spaces $M_{c}^{q}(\Delta_{q}^{m})$, $M_{0}^{q}(\Delta_{q}^{m})$, $M_{p}^{q}(\Delta_{q}^{m})$ and $M_{\infty }^{q}(\Delta_{q}^{m})$. Before proceeding, we recall the definitions of *α*-, *β*- and *γ*-duals.

**Definition 3.2.** *The *α*-, *β*- and *γ*-duals of a subset X∈ω are defined by*


$$X^{\alpha }=\{z=(z_{s})\in \omega :zv=(z_{s}v_{s})\in l_{1}\text{ for all }v\in X\},$$

$$X^{\beta }=\{z=(z_{s})\in \omega :zv=(z_{s}v_{s})\in cs\text{ for all }v\in X\},$$

$$X^{\gamma }=\{z=(z_{s})\in \omega :zv=(z_{s}v_{s})\in bs\text{ for all }v\in X\},$$

*respectively.*

Before proceeding further, we recall certain lemmas from [25] that are necessary for determining the duals. Throughout the paper, let F denotes the family of all finite subsets of $N_{0}$ and $\hat{p}$ be the compliment of *p*, that is, $\frac{1}{\hat{p}}+\frac{1}{p}=1.$

**Lemma 3.1.** $H=(h_{st})\in (c_{0},l_{1})$ *if and only if*

$$\underset{A\in F}{\sup}(\sum_{t=0}^{\infty }|\sum_{s\in A}h_{st}|)<\infty .$$ (5)

**Lemma 3.2.** $H=(h_{st})\in (c_{0},c)$ *if and only if*

$$\underset{s\in N_{0}}{\sup}\sum_{t=0}^{s}|h_{st}|<\infty ,$$ (6)

$$\lim_{s\to \infty }h_{st}\text{ exists for each }t\in N_{0}.$$ (7)

**Lemma 3.3.** $H=(h_{st})\in (c_{0},l_{\infty })$ *if and only if* Eq (6) *holds.*

**Theorem 3.5.** *Define the set* *a*_*1*_(*q*) *by*


a1(q)={z=(zt)∈ω:supA∈F(∑t=0∞|∑s∈A(−1)s−tMt+2(q)−Mt+1(q)Ms(q)Ps−t(q)qs(m+s−t−1s−t)qzs|)<∞}.

*Then* $\left[M_{c}^{q}(\Delta_{q}^{m}){]}^{\alpha }=[M_{0}^{q}(\Delta_{q}^{m}){]}^{\alpha }=a_{1}(q\right)$.

*Proof:* Consider the following equality

zsvs=∑t=0s(−1)s−tMt+2(q)−Mt+1(q)Ms(q)Ps−t(q)qs(m+s−t−1s−t)qzsut=(G(q)u)s, (8)

for all $s\in N_{0}$, where the sequence (*u*_*t*_) is the $M^{q}(\Delta_{q}^{m})$-transform of a sequence $v=(v_{t})$ and the matrix $G(q)=(g_{st}^{q})$ is defined by


gstq={(−1)s−tMt+2(q)−Mt+1(q)Ms(q)Ps−t(q)qs(m+s−t−1s−t)q,0≤t≤s0,t>s.

From equation Eq (8), we realize that $(zv)=(z_{s}v_{s})\in l_{1}$, whenever $v\in M_{0}^{q}(\Delta_{q}^{m})$ if and only if $G(q)u\in l_{1},$ whenever $u\in c_{0}.$ Thus, we deduce that *z* = (*z*_*s*_) belongs to the *α*-dual of the spaces $M_{0}^{q}(\Delta_{q}^{m})$ if and only if the matrix $G(q)\in (c_{0},l_{1}).$ Thus, from Lemma 3.1, we conclude that the *α*-dual of the space $M_{0}^{q}(\Delta_{q}^{m})$ is *a*_1_(*q*). □

**Theorem 3.6.** *Define the sets $a_{2}(q),a_{3}(q)$ and* *a*_*4*_(*q*) *by*


a2(q)={z=(zs)∈ω:∑s=t∞(−1)s−tPs−t(q)qsMt+2(q)−Mt+1(q)Ms(q)(m+s−t−1s−t)qzs exists for each t∈N0},

a3(q)={z=(zs)∈ω:sups∈N0∑t=0s|∑j=ts(−1)j−tPj−t(q)qjMt+2(q)−Mt+1(q)Mj(q)(m+j−t−1j−t)qzj|<∞},

a4(q)={z=(zs)∈ω:lims→∞∑t=0s|∑j=ts(−1)j−tPj−t(q)qjMt+2(q)−Mt+1(q)Mj(q)(m+j−t−1j−t)qzj| exists}.

*Then* $\left[M_{0}^{q}(\Delta_{q}^{m}){]}^{\beta }=a_{2}(q)\cap a_{3}(q\right)$ *and* $[M_{c}^{q}(\Delta_{q}^{m}){]}^{\beta }=a_{2}(q)\cap a_{3}(q)\cap a_{4}(q).$

*Proof:* Consider the following equality

∑t=0sztvt=∑t=0s{∑j=0t(−1)t−jPt−j(q)qtMj+2(q)−Mj+1(q)Mt(q)(m+t−j−1t−j)quj}zt=∑t=0s{∑j=ts(−1)j−tPj−t(q)qjMt+2(q)−Mt+1(q)Mj(q)(m+j−t−1j−t)qzj}ut=(H(q)u)s, (9)

for each $s\in N_{0},$ where the sequence *u* = (*u*_*t*_) is the $M^{q}(\Delta_{q}^{m})$-transform of a sequence $v=(v_{t})$ and the matrix $H(q)=(h_{st}^{q})$ is defined by


hstq={∑j=ts(−1)j−tPj−t(q)qjMt+2(q)−Mt+1(q)Mj(q)(m+j−t−1j−t)qzj,0≤t≤s0,t>s

for all $s,t\in N_{0}.$ From equation Eq (9), we realize that $zv=(z_{s}v_{s})\in cs$, whenever $v=(v_{s})\in M_{0}^{q}(\Delta_{q}^{m})$ if and only if H(q)u∈c whenever $u=(u_{t})\in c_{0}.$ Thus, we deduce that *z* = (*z*_*s*_) belongs to the *β*-dual of the space $M_{0}^{q}(\Delta_{q}^{m})$ if and only if the matrix $H(q)\in (c_{0},c).$ Thus, from Lemma 3.2, we have $\sup_{s\in N_{0}}\sum_{t=0}^{s}|h_{st}^{q}|<\infty$ and $\lim_{s\to \infty }h_{st}^{q}\text{ exists for each }t\in N_{0}.$ Thus, $\left[M_{0}^{q}(\Delta_{q}^{m}){]}^{\beta }=a_{2}(q)\cap a_{3}(q\right)$. □

**Theorem 3.7.** *The *γ*-dual of the spaces* $M_{0}^{q}(\Delta_{q}^{m})$ and $M_{c}^{q}(\Delta_{q}^{m})$ *is a*_3_(*q*).

*Proof:* The proof is similar to the Theorem 3.6 except that Lemma 3.3 is employed instead of Lemma 3.2. □

**Lemma 3.4.** *[*25*–*27*]. The following statements holds true:*

*1.* $H=(h_{st})\in (l_{\infty },l_{\infty })$ *iff*
  $$\underset{s\in N_{0}}{\sup}\sum_{t=0}^{\infty }|h_{st}|<\infty .$$ (10)
*2.* $H=(h_{st})\in (l_{\infty },c)$ *iff*
  $$\;there\;exists\;h_{t}\in C\;such\;that\;\lim_{s\to \infty }h_{st}=h_{t}\;for\;all\;t\in N_{0},$$ (11)
  $$\lim_{s\to \infty }\sum_{t=0}^{\infty }|h_{st}|=\sum_{t=0}^{\infty }|\lim_{s\to \infty }h_{st}|.$$ (12)
*3.* $H=(h_{st})\in (l_{\infty },l_{1})$ *iff*
  $$\underset{A\in F}{\sup}\sum_{t=0}^{\infty }|\sum_{s\in A}h_{st}|<\infty .$$ (13)
*4.* $H=(h_{st})\in (l_{p},l_{\infty })$ *iff*
  $$\underset{s\in N_{0}}{\sup}\sum_{t=0}^{\infty }|h_{st}{|}^{\hat{p}}<\infty ,(1<p<\infty )$$ (14)
  $$\underset{s,t\in N_{0}}{\sup}|h_{st}{|}^{p}<\infty ,(0<p\leq 1).$$ (15)
*5.*
  *(a)* *For* 1<p<∞, $H=(h_{st})\in (l_{p},c)$ *iff* Eq (11) *and* Eq (14) *hold.*
  *(b)* *For 0<p≤1, $H=(h_{st})\in (l_{p},c)$ iff* Eq (11) *and* Eq (15) *hold.*
*6.* $H=(h_{st})\in (l_{p},l_{1})$ *iff*
  $$\underset{A\in F}{\sup}\underset{t\in N_{0}}{\sup}|\sum_{s\in A}h_{st}{|}^{p}<\infty ,\;(0<p\leq 1).$$ (16)
  $$\underset{A\in F}{\sup}\sum_{t=0}^{\infty }|\sum_{s\in A}h_{st}{|}^{\hat{p}}<\infty .,\;(1<p<\infty ).$$ (17)

**Theorem 3.8.** *Define the sets $b_{1}(q),b_{2}(q)$ and* *b*_3_(*q*) *by*


b1(q)={z=(zs)∈ω:supA∈Fsupt∈N0|∑s∈A(−1)s−tPs−t(q)qsMt+2(q)−Mt+1(q)Ms(q)(m+s−t−1s−t)qzs|p<∞},(0<p≤1),

b2(q)={z=(zs)∈ω:supA∈F∑t=0∞|∑s∈A(−1)s−tPs−t(q)qsMt+2(q)−Mt+1(q)Ms(q)(m+s−t−1s−t)qzs|p^<∞},(1<p<∞)

*and*


b3(q)={z=(zs)∈ω:supA∈F∑t=0∞|∑s∈A(−1)s−tPs−t(q)qsMt+2(q)−Mt+1(q)Ms(q)(m+s−t−1s−t)qzs|<∞}.

*Then*

*1.* $[M_{p}^{q}(\Delta_{q}^{m}){]}^{\alpha }=\left\{ \begin{array}{cc} b_{1}(q), & \text{ if }0<p\leq 1\\ b_{2}(q), & \text{ if }1<p<\infty . \end{array}\right.$
*2.* $[M_{\infty }^{q}(\Delta_{q}^{m}){]}^{\alpha }=b_{3}(q).$

*Proof:* From equation Eq (8), we realize that $(zv)=(z_{s}v_{s})\in l_{1}$, whenever $v\in M_{p}^{q}(\Delta_{q}^{m})$ if and only if $G(q)u\in l_{1},$ whenever $u\in l_{p}.$ Thus, we deduce that *z* = (*z*_*p*_) belongs to the *α*-dual of the spaces $M_{p}^{q}(\Delta_{q}^{m})$ if and only if the matrix $G(q)\in (l_{p},l_{1}.)$ Thus, from Lemma 3.4/(vi), we conclude $[M_{p}^{q}(\Delta_{q}^{m}){]}^{\alpha }=\left\{ \begin{array}{cc} b_{1}(q), & \text{ if }0<p\leq 1\\ b_{2}(q), & \text{ if }1<p<\infty . \end{array}\right.$

In a similar manner by utilizing Lemma 3.4/(iii) instead of Lemma 3.4/(vi), we get $\left[M_{\infty }^{q}(\Delta_{q}^{m}){]}^{\alpha }=b_{3}(q\right)$. Hence, the result. □

**Theorem 3.9.** *Define the sets $b_{4}(q),b_{5}(q),b_{6}(q),b_{7}(q)$ by*


b4(q)={z=(zs)∈ω:lims→∞∑j=ts(−1)j−tPj−t(q)qjMt+2(q)−Mt+1(q)Mj(q)(m+j−t−1j−t)qzj exists for each t∈N0},

b5(q)={z=(zs)∈ω:supt,s∈N0|∑j=ts(−1)j−tPj−t(q)qjMt+2(q)−Mt+1(q)Mj(q)(m+j−t−1j−t)qzj|p<∞},(0<p≤1),

b6(q)={z=(zs)∈ω:sups∈N0∑t=0s|∑j=ts(−1)j−tPj−t(q)qjMt+2(q)−Mt+1(q)Mj(q)(m+j−t−1j−t)qzj|p^<∞,

*and*


b7(q)={z=(zs)∈ω:lims→∞∑t=0∞|∑j=ts(−1)j−tPj−t(q)qjMt+2(q)−Mt+1(q)Mj(q)(m+j−t−1j−t)qzj|=∑t=0∞|lims→∞∑j=ts(−1)j−tPj−t(q)qjMt+2(q)−Mt+1(q)Mj(q)(m+j−t−1j−t)qzj|}.

*Then*

*1.* $[M_{p}^{q}(\Delta_{q}^{m}){]}^{\beta }=\left\{ \begin{array}{cc} b_{4}(q)\cap b_{5}(q), & 0<p\leq 1\\ b_{4}(q)\cap b_{6}(q), & 1<p<\infty . \end{array}\right.$
*2.* $[M_{\infty }^{q}(\Delta_{q}^{m}){]}^{\beta }=b_{3}(q)\cap b_{7}(q).$

*Proof:* From equation Eq (9), we realize that $zv=(z_{s}v_{s})\in cs$ whenever $v=(v_{s})\in M_{p}^{q}(\Delta_{q}^{m})$ if and only if H(q)u∈c whenever $u=(u_{s})\in l_{p}.$ This yields that *z* = (*z*_*s*_) belongs to the *β*-dual of the space $M_{p}^{q}(\Delta_{q}^{m})$ if and only if the matrix $H(q)\in (l_{p},c).$ Thus, from Lemma 3.4/(v), we have $[M_{p}^{q}(\Delta_{q}^{m}){]}^{\beta }=\left\{ \begin{array}{cc} b_{4}(q)\cap b_{5}(q), & \text{ if }0<p\leq 1\\ b_{4}(q)\cap b_{6}(q), & \text{ if }1<p<\infty . \end{array}\right.$

In a similar manner by utilizing Lemma 3.4/(ii) instead of Lemma 3.4/(v), we get $[M_{\infty }^{q}(\Delta_{q}^{m}){]}^{\beta }=b_{3}(q)\cap b_{7}(q).$ Hence, the result. □

**Theorem 3.10.** *The following statements hold true:*

*1.* $[M_{p}^{q}(\Delta_{q}^{m}){]}^{\gamma }=\left\{ \begin{array}{cc} b_{5}(q), & \text{ if }0<p\leq 1\\ b_{6}(q), & \text{ if }1<p<\infty . \end{array}\right.$
*2.* $\left[M_{\infty }^{q}(\Delta_{q}^{m}){]}^{\gamma }=b_{6}(q\right)$ *with* $\hat{p}=1.$

## Matrix transformations on the spaces $M_{c}^{q}(\Delta_{q}^{m})$ and $M_{0}^{q}(\Delta_{q}^{m})$

In this section, we determine necessary and sufficient condition for a matrix transformation from the spaces $M_{c}^{q}(\Delta_{q}^{m})$ and $M_{0}^{q}(\Delta_{q}^{m})$ to the spaces $l_{\infty },c,c_{0},l_{1},bs,cs,cs_{0}$. The following theorem is fundamental in our investigation.

**Theorem 4.11.** *Let U be an arbitrary subset of ω. Then*

*1.* $L=(l_{st})\in (M_{0}^{q}(\Delta_{q}^{m});U)⟺$ $A^{\left(s\right)}=(a_{jt}^{\left(s\right)})\in (c_{0},c)$ *and* $A=(a_{st})\in (c_{0},U),$
*2.* $L=(l_{st})\in (M_{c}^{q}(\Delta_{q}^{m});U)⟺$ $A^{\left(s\right)}=(a_{jt}^{\left(s\right)})\in (c,c)$ *and* $A=(a_{st})\in (c,U),$

*where*


ajt(s)={∑i=tj(−1)i−tPi−t(q)qiMt+2(q)−Mt+1(q)Mi(q)(m+i−t−1i−t)qlsi,0≤t≤j0,t>j

*and*

ast=∑i=t∞(−1)i−tPi−t(q)qiMt+2(q)−Mt+1(q)Mi(q)(m+i−t−1i−t)qlsi, (18)

*for all* $s,t\in N_{0}.$

*Proof:* The proof is similar to the proof of Theorem 4.1 of [7] and hence is omitted. □

Now, by using the results presented in [25] together with Theorem 4.11, we obtain the following results.

**Corollary 4.1.** *The following statements hold:*

*1.* $L=(l_{st})\in (M_{0}^{q}(\Delta_{q}^{m});l_{\infty })$ *iff*
  $$\underset{j\in N_{0}}{\sup}\sum_{t=0}^{\infty }|a_{jt}^{\left(s\right)}|<\infty ,$$ (19)
  $$\lim_{j\to \infty }a_{jt}^{\left(s\right)}\;exists\;for\;all\;t\in N_{0}\;hold\;and\;$$ (20)
  $$\underset{s\in N_{0}}{\sup}\sum_{t=0}^{s}|a_{st}|<\infty ,$$ (21)
*also hold.*
*2.* $L=(l_{st})\in (M_{0}^{q}(\Delta_{q}^{m});c)$ *iff* Eq (19) *and* Eq (20) *hold, and*
  $$\underset{s\in N_{0}}{\sup}\sum_{t=0}^{\infty }|a_{st}|<\infty ,$$ (22)
  $$\lim_{s\to \infty }a_{st}\;exists\;for\;all\;t\in N_{0},$$ (23)
*also hold.*
*3.* $L=(l_{st})\in (M_{0}^{q}(\Delta_{q}^{m});c_{0})$ *ff* Eq (19) *and* Eq (20) *hold, and* Eq (21) *and*
  $$\lim_{s\to \infty }a_{st}=0\;for\;all\;t\in N_{0}$$ (24)
*also hold.*
*4.* $L=(l_{st})\in (M_{0}^{q}(\Delta_{q}^{m});l_{1})$ *iff* Eq (19) *and* Eq (20) *hold, and*
  $$\underset{A\in F}{\sup}\sum_{t=0}^{\infty }|\sum_{s\in A}a_{st}|<\infty ,$$ (25)
*also hold.*
*5.* $L=(l_{st})\in (M_{0}^{q}(\Delta_{q}^{m});bs)$ *iff* Eq (19) *and* Eq (20) *hold, and*
  $$\underset{s\in N_{0}}{\sup}\sum_{t=0}^{\infty }|\sum_{j=0}^{s}a_{jt}|<\infty ,$$ (26)
*also hold.*
*6.* $L=(l_{st})\in (M_{0}^{q}(\Delta_{q}^{m});cs)$ *iff* Eq (19) *and* Eq (20) *hold, and* Eq (26) *and*
  $$\sum_{s=0}^{\infty }a_{st}\;converges\;for\;all\;t\in N_{0},$$ (27)
*also hold.*
*7.* $L=(l_{st})\in (M_{0}^{q}(\Delta_{q}^{m});cs_{0})$ *iff* Eq (19) *and* Eq (20) *hold, and* Eq (26) *and*
  $$\sum_{s=0}^{\infty }a_{st}=0\;for\;all\;t\in N_{0},$$ (28)
*also hold.*

**Corollary 4.2.** *The following statements hold:*

*1.* $L=(l_{st})\in (M_{c}^{q}(\Delta_{q}^{m});l_{\infty })$ *iff* Eq (19) *and* Eq (20) *hold, and*
  $$\lim_{j\to \infty }\sum_{t=0}^{\infty }a_{jt}^{\left(s\right)},$$ (29)
  *hold and* Eq (22) *also hold.*
*2.* $L=(l_{st})\in (M_{c}^{q}(\Delta_{q}^{m});c)$ *iff* Eq (19), Eq (20) *and* Eq (29) *hold, and* Eq (21), Eq (23) *and*
  $$\lim_{s\to \infty }\sum_{t=0}^{s}a_{st}\;exists$$ (30)
*also hold.*
*3.* $L=(l_{st})\in (M_{c}^{q}(\Delta_{q}^{m});c_{0})$ *iff* Eq (19), Eq (20) *and* Eq (29) *hold, and* Eq (21), Eq (24) *and*
  $$\lim_{s\to \infty }\sum_{t=0}^{s}a_{st}=0,$$ (31)
*also hold.*
*4.* $L=(l_{st})\in (M_{c}^{q}(\Delta_{q}^{m});l_{1})$ *iff* Eq (19), Eq (20) *and* Eq (29) *hold, and* Eq (25) *also hold.*
*5.* $L=(l_{st})\in (M_{c}^{q}(\Delta_{q}^{m});bs)$ *iff* Eq (19), Eq (20) *and* Eq (29) *hold, and Eq (*26*) also hold.*
*6.* $L=(l_{st})\in (M_{c}^{q}(\Delta_{q}^{m});cs)$ *iff* Eq (19), Eq (20) *and* Eq (29) *hold, and* Eq (26), Eq (27) *and*
  $$\sum_{s=0}^{\infty }\sum_{t=0}^{\infty }a_{st}\;converges$$ (32)
*also hold.*
*7.* $L=(l_{st})\in (M_{c}^{q}(\Delta_{q}^{m});cs_{0})$ *iff* Eq (19), Eq (20) *and* Eq (29) *hold, and* Eq (26), Eq (27) *and*
  $$\sum_{s=0}^{\infty }\sum_{t=0}^{\infty }a_{st}=0$$ (33)
*also hold.*

## Compact operators on the spaces $M_{0}^{q}(\Delta_{q}^{m})$

Let *X* and *Y* be Banach spaces. Let *U*_*X*_ denotes the open ball in the space *X* and B(X,Y) be the set of all bounded linear operators T:X→Y, is a Banach space with the norm given by $\|T\|=\sup_{x\in U_{X}}\|T(x){\|}_{Y}$. Further, we denote $\|z{\|}_{X}^{*}=\sup_{u\in U_{X}}|\sum_{t=0}^{\infty }z_{t}u_{t}|,$ provided the expression on the right hand side exists and is finite [28]. A linear operator T:X→Y is said to be compact if the domain of *T* is all of *X* and for every bounded sequence $(x_{n})\in X,$ the sequence (*T*(*x*_*n*_)) has a subsequence which converges in *Y*.

Let *M* be a bounded set in a metric space *X*. Then the Hausdorff measure of non-compactness(Hmnc) of *M* is defined by


$$\chi (M)=\inf\{\epsilon >0:M\subset \cup_{k=0}^{n}B(z_{k},r_{k}),z_{k}\in X,r_{k}<\epsilon (k=0,1,2,\ldots ,n),n\in N_{0}\},$$

where $B(z_{k},r_{k})$ is the open ball centered at *z*_*k*_ and radius *r*_*k*_ for each k=0,1,2,…,n. The operator *T* is compact if and only if $\|T{\|}_{\chi }=0,$ where $\|T{\|}_{\chi }$ denotes the **Hmnc** of *T* and is defined by $\|T{\|}_{\chi }=\chi (T(U_{X})).$ For more details about **Hmnc**, one can see [29] and references therein. The **Hmnc** of a linear operator plays a role to characterize the compactness of an operator between BK spaces. For the relevant literature, see [30–32].

Let *X* and *Y* be any two BK spaces, then every matrix P∈(X,Y) defines a linear operator $T_{P}\in B(X,Y),$ where $T_{P}z=Pz$ for all z∈X (see Theorem 3.2.4 of [33]). Moreover, if X⊃Ψ is a BK-space then $\|T_{P}\|=\|P{\|}_{\left(X,Y\right)}=\sup_{n\in N_{0}}\|P{\|}_{X}^{*}<\infty$ (see Theorem 1.23 of [29]).

Let $z=(z_{t})\in \omega$ and define a sequence $v=(v_{t})$ as


vt=∑s=t∞(−1)s−tPs−t(q)qsMt+2(q)−Mt+1(q)Ms(q)(m+s−t−1s−t)qzs

for all $t\in N_{0}.$

**Lemma 5.5.** *[*34*]* $l_{\infty }^{\beta }=c^{\beta }=c_{0}^{\beta }=l_{1}$ *and* $\|z{\|}_{X}^{*}=\|z{\|}_{l_{1}}$ *for* $X\in \{l_{\infty },c,c_{0}\}.$

**Lemma 5.6.** *[*29*] Let $P_{n}:c_{0}\to c_{0}$ be the operator defined by $P_{n}(x)=(x_{0},x_{1},x_{2},\ldots ,x_{n},0,0,\ldots )$ for all $x=(x_{n})\in c_{0}$. Then for any bounded subset M in *c*_*0*_, we have*


$$\chi (M)=\lim_{n\to \infty }(\underset{x\in M}{\sup}\|(I-P_{n})(x){\|}_{c_{0}}),$$

*where I is the identity operator on *c*_*0*_.*

**Lemma 5.7.** *Let $z=(z_{t})\in [M_{0}^{q}(\Delta_{q}^{m}){]}^{\beta }.$ Then $v=(v_{t})\in l_{1}$ and*

$$\sum_{t}z_{t}x_{t}=\sum_{t}v_{t}y_{t}$$ (34)

*for all* $x=(x_{t})\in M_{0}^{q}(\Delta_{q}^{m}).$

**Lemma 5.8.** $\|z{\|}_{M_{0}^{q}(\Delta_{q}^{m})}^{*}=\sum_{t}|v_{t}|<\infty$ *for all* $z=(z_{t})\in [M_{0}^{q}(\Delta_{q}^{m}){]}^{\beta }.$

*Proof:* Let $z=(z_{t})\in [M_{0}^{q}(\Delta_{q}^{m}){]}^{\beta }.$ Then, by Lemma 5.7, we have $v=(v_{t})\in l_{1}$ and Eq (34) holds. Since, $\|x{\|}_{M_{0}^{q}(\Delta_{q}^{m})}=\|y{\|}_{c_{0}}$ holds, we get $x\in U_{M_{0}^{q}(\Delta_{q}^{m})}$ if and only if $y\in U_{c_{0}}.$ Hence, we conclude that


$$\|z{\|}_{M_{0}^{q}(\Delta_{q}^{m})}^{*}=\underset{x\in U_{M_{0}^{q}(\Delta_{q}^{m})}}{\sup}|\sum_{t}z_{t}x_{t}|=\underset{y\in U_{c_{0}}}{\sup}|\sum_{t}v_{t}y_{t}|=\|v{\|}_{c_{0}}^{*}.$$

From Lemma 5.5, it follows that $\|z{\|}_{M_{0}^{q}(\Delta_{q}^{m})}^{*}=\|v{\|}_{c_{0}}^{*}=\|v{\|}_{l_{1}}=\sum_{t}|v_{t}|.$ □

**Lemma 5.9.** *Let X∈ω and *L* = (*l*_*st*_) be an infinite matrix. If $L\in (M_{0}^{q}(\Delta_{q}^{m}),X)$, then $A\in (c_{0},X)$ and *Lx* = *Ay* for all $x\in M_{0}^{q}(\Delta_{q}^{m}),$ where L and A are related by the relation* Eq (18).

*Proof:* The proof of this Lemma follows from Lemma 5.7. □

**Lemma 5.10.** $\|T_{L}\|=\|L{\|}_{\left(M_{0}^{q}(\Delta_{q}^{m}),U\right)}=\sup_{s\in N_{0}}(\sum_{t}|a_{st}|)<\infty$ holds for $L\in (M_{0}^{q}(\Delta_{q}^{m}),U),$ where $U\in \{c_{0},c,l_{\infty }\}.$

**Lemma 5.11.** *[*35*] Let X⊃Ψ be a BK-space. Then the following statements hold.*

*1.* $L\in (X,l_{\infty }),$ *then* $0\leq \|T_{L}{\|}_{\chi }\leq {lim sup}_{s}\|L_{s}{\|}_{X}^{*}$ *and T*_*L*_ *is compact if and only if* $\lim_{s\to \infty }\|L_{s}{\|}_{X}^{*}=0.$
*2.* $L\in (X,c_{0}),$ *then* $\|T_{L}{\|}_{\chi }={lim sup}_{s}\|L_{s}{\|}_{X}^{*}$ *and T*_*L*_ *is compact if and only if* $\lim_{s\to \infty }\|L_{s}{\|}_{X}^{*}\;=\;0.$
*3.* *If X has AK or* $X=l_{\infty }$ *and* L∈(X,c), *then* $\frac{1}{2}{lim sup}_{s}\|L_{s}$ − $l{\|}_{X}^{*}\leq \|T_{L}\|\chi \leq {lim sup}_{s}\|$ *L*_*s*_ − $l{\|}_{X}^{*}$, *where l* = (*l*_*t*_) *and* $l_{t}=\lim_{s}l_{st}$ *for each* $t\in N_{0}.$

**Lemma 5.12.** *[*35*] Let X⊃Ψ be a BK-space. If $L\in (X,l_{1}),$ then*


$$\lim_{n}(\underset{A\in F_{n}}{\sup}\|\sum_{s\in A}L_{s}{\|}_{X}^{*})\leq \|T_{L}{\|}_{\chi }\leq 4\lim_{n}(\underset{A\in F_{n}}{\sup}\|\sum_{s\in A}L_{s}{\|}_{X}^{*})$$

*and *T*_*L*_ is compact if and only if $\lim_{n}(\sup_{A\in F_{n}}\|\sum_{s\in A}L_{s}{\|}_{X}^{*})=0,$ where $F_{n}$ is the sub-collection of F consisting of all nonempty and finite subsets of N with elements that are greater than n.*

**Theorem 5.12.**

*1.* *If* $L\in (M_{0}^{q}(\Delta_{q}^{m}),l_{\infty }),$ *then* $0\leq \|T_{L}{\|}_{\chi }\leq {lim sup}_{s}\sum_{s}|a_{st}|$ *holds.*
*2.* If $L\in (M_{0}^{q}(\Delta_{q}^{m}),c),$ *then* $\frac{1}{2}{lim sup}_{s}\sum_{t}|a_{st}-l_{t}|\leq \|T_{L}{\|}_{\chi }\leq {lim sup}_{s}\sum_{t}|a_{st}-l_{t}|$ *holds*.
*3.* *If* $L\in (M_{0}^{q}(\Delta_{q}^{m}),c_{0}),$ *then* $\left\|T_{L}{\|}_{\chi }={lim sup}_{s}\sum_{t}|a_{st}\right|$ *holds*.
*4.* If $L\in (M_{0}^{q}(\Delta_{q}^{m}),l_{1}),$ *then* $\lim_{n}\|L{\|}_{\left(M_{0}^{q}(\Delta_{q}^{m}),l_{1}\right)}^{\left(n\right)}\leq \|T_{L}{\|}_{\chi }\leq 4\lim_{n}\|L{\|}_{\left(M_{0}^{q}(\Delta_{q}^{m}),l_{1}\right)}^{\left(n\right)}$ *holds, where*
  $$\|L{\|}_{\left(M_{0}^{q}(\Delta_{q}^{m}),l_{1}\right)}^{\left(n\right)}=\underset{A\in F_{n}}{\sup}(\sum_{t}|\sum_{s\in A}a_{st}|{)}_{X}^{*}\;(n\in N_{0}).$$

*Proof:*

1. Let $L\in (M_{0}^{q}(\Delta_{q}^{m}),l_{\infty }).$ Since the series $\sum_{t=1}^{\infty }l_{st}x_{t}$ converges for each $s\in N_{0},$ we have $L_{s}\in [M_{0}^{q}(\Delta_{q}^{m}){]}^{\beta }.$ From Lemma 5.8, we have $\|L_{s}{\|}_{M_{0}^{q}(\Delta_{q}^{m})}^{*}=\|A_{s}{\|}_{c_{0}}^{*}=\|A_{s}{\|}_{l_{1}}=\sum_{t}|a_{st}|,$ for each $s\in N_{0}.$ By using Lemma 5.11 (i), we deduce that $0\leq \|T_{L}{\|}_{\chi }\leq {lim sup}_{s}\sum_{s}|a_{st}|.$
2. Let $L\in (M_{0}^{q}(\Delta_{q}^{m}),c).$ By Lemma 5.9, we have $A\in (c_{0},c).$ Hence, from Lemma 5.11 (iii), we have $\frac{1}{2}{lim sup}_{s}\|A_{s}\;-\;a{\|}_{c_{0}}^{*}\leq \|T_{L}\|\chi \leq {lim sup}_{s}\|A_{s}\;-\;a{\|}_{c_{0}}^{*}$, where *a* = (*a*_*t*_) and $a_{t}=\lim_{s}a_{st}$ for each $t\in N_{0}.$ Moreover, Lemma 5.7 implies that $\left\|A_{s}\;-\;a{\|}_{c_{0}}^{*}=\|A_{s}-a{\|}_{l_{1}}=(\sum_{t}|a_{st}\;-\;a_{t}|\right)$ for each $s\in N_{0}.$
3. Let $L\in (M_{0}^{q}(\Delta_{q}^{m}),c).$ Since, $\|L_{s}{\|}_{M_{0}^{q}(\Delta_{q}^{m})}^{*}=\|A_{s}{\|}_{c_{0}}^{*}=\|A_{s}{\|}_{l_{1}}=\sum_{t}|a_{st}|,$ for each $s\in N_{0},$ from Lemma 5.11(ii), we have $\left\|T_{L}{\|}_{\chi }={lim sup}_{s}\sum_{t}|a_{st}\right|$.
4. Let $L\in (M_{0}^{q}(\Delta_{q}^{m}),l_{1}).$ By Lemma 5.9, we have $A\in (c_{0},l_{1}).$ From Lemma 5.12, it follows that
  $$\lim_{n}(\underset{A\in F_{n}}{\sup}\|\sum_{s\in A}A_{s}{\|}_{c_{0}}^{*})\leq \|T_{L}{\|}_{\chi }\leq 4\lim_{n}(\underset{A\in F_{n}}{\sup}\|\sum_{s\in A}A_{s}{\|}_{c_{0}}^{*}).$$
  Moreover, Lemma 5.5 implies that $\left\|\sum_{s\in F}A_{s}{\|}_{c_{0}}^{*}=\|\sum_{s\in F}A_{s}{\|}_{l_{1}}=(\sum_{t}|\sum_{s\in F}a_{st}|\right)$.

□

**Corollary 5.3.**

*1.* *T*_*L*_ *is compact for* $L\in (M_{0}^{q}(\Delta_{q}^{m}),l_{\infty })$ *if* $\lim_{s}\sum_{t}|a_{st}|=0.$
*2.* *T*_*L*_ *is compact for* $L\in (M_{0}^{q}(\Delta_{q}^{m}),c)$ *if and only if* $\lim_{s}\sum_{t}|a_{st}-a_{t}|=0.$
*3.* *T*_*L*_ *is compact for* $L\in (M_{0}^{q}(\Delta_{q}^{m}),c_{0})$ *if and only if* $\lim_{s}\sum_{t}|a_{st}|=0.$
*4.* *T*_*L*_ *is compact for* $L\in (M_{0}^{q}(\Delta_{q}^{m}),l_{1})$ *if and only if* $\lim_{n}\|L{\|}_{\left(M_{0}^{q}(\Delta_{q}^{m}),l_{1}\right)}^{\left(n\right)}=0,$ *where* $\|L{\|}_{\left(M_{0}^{q}(\Delta_{q}^{m}),l_{1}\right)}^{\left(n\right)}=\sup_{A\in F_{n}}(\sum_{t}|\sum_{s\in A}a_{st}|).$

## Conclusion

Due to the vast application of quantum calculus, Motzkin numbers and difference operator in various mathematical and scientific disciplines, we have constructed generalized *q*-difference Motzkin sequence spaces $M_{c}^{q}(\Delta_{q}^{m})$, $M_{0}^{q}(\Delta_{q}^{m})$, $M_{p}^{q}(\Delta_{q}^{m})$ and $M_{\infty }^{q}(\Delta_{q}^{m})$ and explore their topological properties. We determine Schauder bases for $M_{c}^{q}(\Delta_{q}^{m})$ and $M_{0}^{q}(\Delta_{q}^{m})$ and compute *α*-, *β*- and *γ*-duals of the newly defined spaces. Further, we characterize some matrix mappings from the spaces $M_{0}^{q}(\Delta_{q}^{m})$ and $M_{c}^{q}(\Delta_{q}^{m})$ to the spaces $l_{\infty },c,c_{0},l_{1},bs,cs,cs_{0}$. Lastly, compact operators are characterized on the spaces $M_{0}^{q}(\Delta_{q}^{m})$.

## References

[1] Stanojević I. The measure of noncompactness of matrix transformations on the spaces $c^{p}(\Delta )$ and $c_{\infty }^{p}(\Delta )$ (1<p<∞). Mat Vesnik. 2005;57:65–78.

[2] Malkowsky E. Modern functional analysis in the theory of sequence spaces and matrix transformations. Jordan J Math Stat. 2008;1:1–29.

[3] Choudhary A, Raj K, Mursaleen M. Compact operators on spaces of binomial fractional difference sequences. Math Sci. 2022;16:79–85.

[4] Devletli U, Kara MI. New Banach Sequence Spaces Defined by Jordan Totient Function. Commun Adv Math Sci. 2023;6:211–25.

[5] Jena D, Dutta S. Fractional ordered Euler Riesz difference sequence spaces. Proyecciones (Antofagasta). 2023;42:1355–72.

[6] Karakas M, Dağlı MC. Some topologic and geometric properties of new Catalan sequence spaces. Adv Oper Theory. 2023;8(1). doi: 10.1007/s43036-022-00243-9

[7] Kirişçi M, Başar F. Some new sequence spaces derived by the domain of generalized difference matrix. Comput Math Appl. 2010;60:1299–309.

[8] Raj K, Choudhary A, Mursaleen M. Bounds for the operator norm on weighted Ces‘aro fractional difference sequence spaces. Sahand Commun Math Anal. 2023;20:19–34.

[9] Raj K, Devi M. Almost convergent sequence spaces defined by Nörlund matrix and generalized difference matrix. Advances in Mathematical Analysis and its Applications. 2022;57–70.

[10] Jackson F. On q-functions and certain difference operator. Trans Roy Soc Edin. 1909;46:253–81.

[11] Demiriz S, Sahin A. q-Cesàro sequences spaces derived by q-analogues. Adv Math. 2016;5:97–110.

[12] Yaying T, Hazarika B, Mursaleen M. On sequence space derived by the domain of q-Cesàro matrix in *l*_*p*_ space and the associated operator ideal. J Math Anal Appl. 2021;493(1):124453.

[13] Yaying T, Hazarika B, Mursaleen M. On generalized (p,q)-Euler matrix and associated sequence spaces. J Funct Spaces. 2021;2021.

[14] Atabey Kİ, Çınar M, Et M. q-Fibonacci sequence spaces and related matrix transformations. J Appl Math Comput. 2022;69(2):2135–54. doi: 10.1007/s12190-022-01825-9

[15] Ellidokuzoglu HB, Demiriz S. On some generalized q-difference sequence spaces. AIMS Math. 2023;8:18607–17.

[16] Alotaibi A, Yaying T, Mohiuddine SA. Sequence spaces and spectrum of q-difference operator of second order. Symmetry. 2022;14(6):1155.

[17] Yaying T, Hazarika B, Et M. On some sequence spaces via q-pascal matrix and its geometric properties. Symmetry. 2023;15(9):1659. doi: 10.3390/sym15091659

[18] Yaying T, Hazarika B, Chandra Tripathy B, Mursaleen M. The spectrum of second order quantum difference operator. Symmetry. 2022;14(3):557. doi: 10.3390/sym14030557

[19] Yaying T, Hazarika B, Mursaleen M. Cesàro sequence spaces via (p,q)-calculus and compact matrix operators. J Anal. 2022;30:1535–53.

[20] Ayman Mursaleen M, Serra-Capizzano S. Statistical convergence via q-calculus and a Korovkin’s type approximation theorem. Axioms. 2022;11(2):70. doi: 10.3390/axioms11020070

[21] Kac V, Cheung P. Quantum calculus. New York, Berlin, Heidelberg: Springer-Verlag. 2001.

[22] Donaghey R, Shapiro LW. Motzkin numbers. Journal of Combinatorial Theory, Series A. 1977;23(3):291–301. doi: 10.1016/0097-3165(77)90020-6

[23] Erdem S, Demiriz S, Sahin A. Motzkin sequence spaces and motzkin core. Numer Funct Anal Optim. 2024;45(4–6):283–303.

[24] Wilansky A. Summability through functional analysis. North-Holland. 1984.

[25] Stieglitz M, Tietz H. Matrixtransformationen von Folgenrumen Eine Ergebnisbersicht. Math Z. 1977;154(1):1–16. doi: 10.1007/bf01215107

[26] Grosseerdmann KG. Matrix transformations between the sequence spaces of Maddox. J Math Anal Appl. 1993;180:223–38.

[27] Lascarides CG, Maddox IJ. Matrix transformations between some classes of sequences. Proc Camb Phil Soc. 1970;68:99–104.

[28] Djolović I, Malkowsky E. A note on Fredholm operators on $(c_{0}{)}_{T}$. Appl Math Lett. 2009;22:1734–9.

[29] Malkowsky E, Rakocevic V. An introduction into the theory of sequence spaces and measure of noncompactness. Zb Rad(Beogr). 2000;9:143–234.

[30] Basarir M, Kara EE. On compact operators on the Riesz B(m)-difference sequence spaces. Iran J Sci Technol Trans A Sci. 2011;35:279–85.

[31] Başarir M, Kara EE. On some difference sequence spaces of weighted means and compact operators. Ann Funct Anal. 2011;2:114–29.

[32] Dağlı MC, Yaying T. Some results on matrix transformation and compactness for fibonomial sequence spaces. Acta Sci Math (Szeged). 2023;89(3–4):593–609. doi: 10.1007/s44146-023-00087-6

[33] Mursaleen M, Basar F. Sequence Spaces, Topic in Modern Summability Theory. CRC Press. 2020.

[34] Malafosse BD. The Banach algebra B(X), where X is a BK space and applications. Mat Vesnik. 2005;57:41–60.

[35] Mursaleen M, Noman AK. Compactness by the Hausdorff measure of noncompactness. Nonlinear Analysis: Theory, Methods & Applications. 2010;73(8):2541–57. doi: 10.1016/j.na.2010.06.030

